# Active video gaming in patients with renal transplant: a pilot study

**DOI:** 10.1186/2047-1440-3-15

**Published:** 2014-08-09

**Authors:** Dorothy Wei Yun Wang, Laura L Sills, Sara B MacDonald, Ziv Maianski, Ian Alwayn

**Affiliations:** 1 Department of Surgery, Multi-Organ Transplant Program, Dalhousie University, Halifax, Canada; 2 Department of Physiotherapy, Dalhousie University, Halifax, Canada; 3 QEII Health Sciences Center, Office 6–291 Victoria Building, 1276 South Park Street, B3H 2Y9 Halifax, NS, Canada

**Keywords:** Transplantation, Renal transplantation, Active video gaming, Xbox, Rehabilitation, Physical activity, Functional status, Quality of life

## Abstract

**Background:**

Patients with renal transplant are at higher risk of mortality from cardiovascular disease (CVD) compared with the general population. Physical activity has been shown to reduce the risk of CVD mortality in these patients. Unfortunately, barriers such as the harsh Canadian climate prevent patients from engaging in and harvesting the health benefits of physical activity. This pilot study explored active video gaming (AVG) as a way for patients with renal transplant to obtain physical activity and examined its effect on their functional status and quality of life (QOL).

**Main text:**

We recruited nine patients for an 8-week prospective pilot study. All patients received a Microsoft Xbox 360™ video gaming console, a Microsoft Kinect™ sensor, and the video game *Your Shape Fitness Evolved 2012*. Assessment of each participant before and after the intervention included blood pressure measures, a 6-minute walk test, and the Godin Leisure Time Questionnaire (GLTQ). We analyzed all nine patients at the end of the 8-week study period, and found no changes in blood pressure or GLTQ scores. However, there was a significant increase in the 6-minute walk distance (*P* = 0.022), which represented a consistent increase for most patients (correlation = 0.977). In addition, participants over the age of 45 years (n = 4) were more likely to use the AVG system (*P* = 0.042).

**Conclusion:**

AVG has the potential to improve the functional status in patients with renal transplant. Further research is required to corroborate the full health benefits of AVG in this patient population.

## Background

Renal transplantation has significantly improved survival rates in patients with chronic kidney disease. However, renal transplant recipients (RTRs) are three to five times more likely to develop and die from cardiovascular disease (CVD) compared with the general population [1] as a result of compromised kidney function [2,3], risk factors such as diabetes and hypertension [4], and long-term use of immunosuppressive mediations with obesogenic side effects [5,6].

It is well established that physical activity reduces the risk of morbidity and mortality from CVD by improving health-related fitness and improving quality of life (QOL) [7,8]. Despite the health benefits of physical activity, candidates for renal transplantation are often physically inactive because of compromised exercise tolerance, musculoskeletal deterioration [7-11] and barriers such as lack of motivation and interest or false beliefs about physical activity. In Canada, the harsh climate imposes further restrictions in outdoor activity [9].

Recently, video games designed to promote physical activity have been introduced as a novel approach to physical fitness and weight loss [12-14]. However, there have not been any studies assessing he impact of active video gaming (AVG) on QOL and cardiovascular health of transplant patient populations. This pilot study aimed to examine the impact of AVG as a form of physical activity on the functional status and QOL of patients with renal transplant.

## Main text

We designed an 8-week prospective pilot study involving AVG as the intervention. This study was approved by the Research Ethics Board of our institution.

Nine adult patients with renal transplant were randomly selected from the Renal Transplant Clinic at the QEII Health Sciences Centre (Halifax, Canada), between June and December 2012. They were at a minimum of 6 months post-transplant, with a functioning graft. Exclusion criteria included significant cardiopulmonary dysfunction, peritransplant complications, photosensitivity, peripheral vascular disease, and no or limited internet access.

At baseline and at the completion of the intervention blood pressure (BP) was measured, and participants completed a 6-minute walk test (6MWT) and the Godin Leisure Time Questionnaire (GLTQ). The 6MWT was performed in accordance with standard protocol [15].

Participants were given an AVG console and gaming/exercise software for the duration of the study. The AVG console included a Microsoft Xbox 360™ video gaming console and Kinect™ (Microsoft Corporation, Mississauga, Ontario, Canada), which is a motion sensor that enables the user to interact with the gaming system using gestures. Participants were instructed to exercise with the game software for a minimum of 30-minute sessions on three separate days per week for the duration of the study. The game used in this study was *Your Shape Fitness Evolved 2012* (Ubisoft) (Ubisoft Canada Inc., Montreal, Quebec, Canada), which is an exercise training program including games, aerobic and toning workouts, and dance classes. We asked participants to focus primarily on aerobic exercises.

Patient characteristics are shown in Table 1. Out of the nine participants, two were students and three were retired. All study participants were able to physically complete the 6MWT without interruptions. Two patients had received their second renal transplant. One patient withdrew from the study, as they did not have sufficient time to start the AVG exercise program by the end of the study at 8 weeks. One patient did not complete a post-intervention 6MWT. All patients were receiving treatments for comorbidities.

**Table 1:** **Patient characteristics**^**a**^

**Characteristic**	**Result**
Patients, n	9
Mean age, years	47 ± 17
Gender (male/female)	7/2
Body mass index	28 ± 5
Current smoker, %	11 (1 participant)
Comorbidities, %:	
Hypertension	100%
Diabetes	33%
High cholesterol	56%
Time since transplant, days	336 ± 138
Time on dialysis, days	695 ± 553
Retired, %	33

^a^Data are mean ± SD unless otherwise indicated.

**Figure 1:**
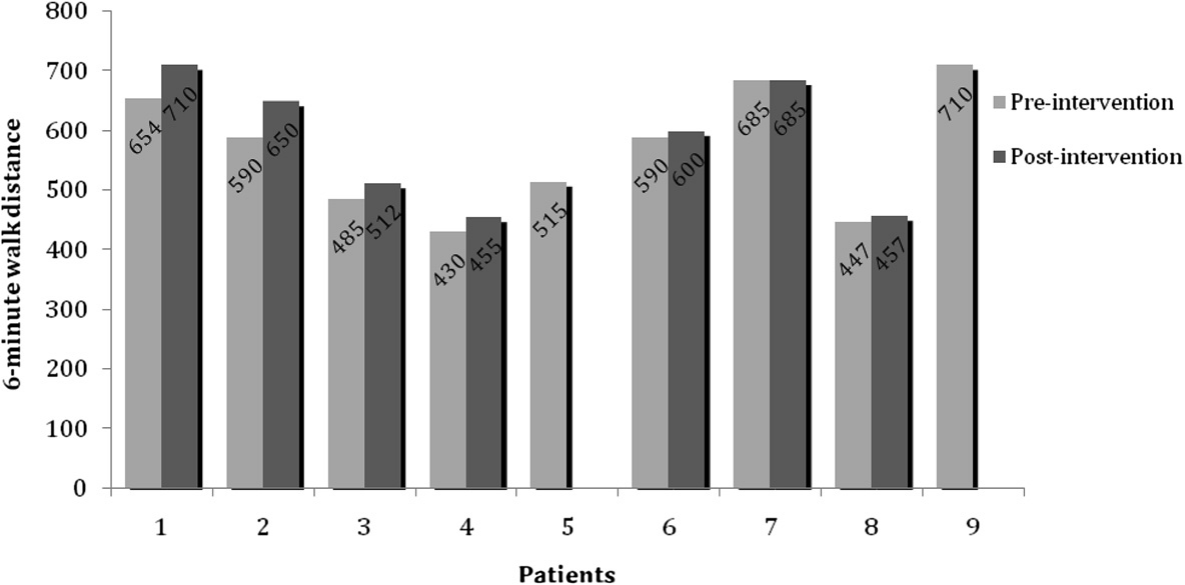
**Six-minute walk test (6MWT) results before and after the intervention.** The 6 minute walk test results of individual patients before (grey bars) and after (black bars) the intervention are shown. Two patients (5 and 9) declined to do the 6-MWTafter the 8-week period because of limited use of the AVG system. Data values are shown above the bars.

The mean 6-minute walk distance (6MWD) was 567 ± 103 before and and 581 ± 107 meters after the intervention. There was a significant increase in the 6MWD post-intervention (*P* = 0.022), which represented a consistent increase for most patients (correlation = 0.977) (Figure 1). Specifically, six of seven participants who completed both pre-intervention and post-intervention 6MWT demonstrated an improvement in their 6MWD. These participants had used their AVG system to exercise at least once. One participant who used the AVG system for 3 days showed no change in 6MWD. There were no statistical differences in BP measures and GLTQ scores as assessed by Student’s paired *t*-test (Table 2, Table 3). There was no correlation between the number of days the AVG was used and the quantitative improvement in the 6MWD (*P* = 0.647; R^2^ = 0.045) as assessed by linear regression. Participants over the age of 45 years (n = 4) were more likely to use the AVG system (*P* = 0.042) as determined by two-tailed Student’s *t*-test. The mean number of days for which participants used the AVG exercise program was 8.4 days. Only one participant achieved the recommended amount of exercise on the AVG system of a minimum of3 days a week of 30-minute sessions for a total of 8 weeks (24 days).

**Table 2:** Blood pressure measures before and after the intervention

**Blood pressure**	**Pre-intervention**^**a**^	**Post-intervention**^**a**^
Systolic	131 ± 15	138 ± 13
Diastolic	82 ± 4	82 ± 8

^a^Data are mean ± SD.

**Table 3:** Godin Leisure Time Exercise questionnaire scores

**Patient**	**Score**	
	**Pre-intervention**	**Post-intervention**
1	56	48
2	55	40
3	25	40
4	6	19
5	12	5
6	14	11
7	30	57
8	0	16
9	54	87
Mean ± SD	28 ± 22	36 ± 26

Physical activity addresses a number of risk factors have been shown to contribute to the increased incidence of and morality from CVD seen in RTRs. Patients needing renal transplantation have compromised cardiovascular health as a result of the decreased glomerular filtration rate (GFR) and increased proteinuria that characterizes chronic kidney disease [2,3]. This is in addition to the large number of common risk factors shared by both chronic kidney disease (CKD) and CVD, such as diabetes, hypertension, and greater age [4]. Moreover, long-term exposure to immunosuppressive medications post-transplantation further increases the risk of CVD. Corticosteroids often result in weight gain [5], and some calcineurin inhibitors are associated with new-onset diabetes [6]. Regular exercise is associated with better GFR [5], health-related fitness [7], and peak oxygen consumption [8] in RTRs. Physical activity also has an important role in maintaining musculoskeletal health and function, and can address issues such as muscle wasting, which can result from both CKD and immunosuppression with corticosteroids, as well as corticosteroid-induced osteoporosis [5,10]. Other benefits of being physically active include improved psychological wellbeing, quality of life, and self-reported function [7,8,11].

Compared with traditional video games, where the participant is stationary, AVG requires the participant to physically move in order to engage the game via a motion sensor. As a form of physical activity, AVG stimulates light to moderate intensity activity in adults and adolescents [12]. Recent research in pediatric and adolescent populations showed AVG to be effective in decreasing body mass index and percentage body fat [12,13]. For RTRs, AVG can increase motivation for exercise with its fun appeal and the various types of physical activity that come with the gaming software, such as warm-up games and dance classes. In addition, it can be an ideal option for indoor exercise in Canada, given the cold climate for the majority of the year. One patient in our study stated that he greatly enjoyed being able to exercise in the comfort of his own home.

The 6MWT is a validated tool to assess the functional status in various chronic diseases including coronary artery disease, COPD and cancer [16-19]. Specifically, it has been found to correspond to patients’ self-reported physical function as well as to clinical measures such as maximum oxygen consumption, forced expiratory volume in 1 second, and forced vital capacity [17,18]. In addition, it has also been found to have good clinical prognostic predictability in patients with end-stage renal diseae [19]. The 6MWD in healthy patients was examined in a study by Casanova *et al*., involving 444 healthy adults aged 40 to 80 years in seven countries. They reported the 6MWD to range from 380 to 782 m, with a mean 6MWD of 571 m [20]. Although there are no established values for subsets of patients with specific chronic conditions, the 6MWD is often part of the overall assessment of the effectiveness of exercise and rehabilitation programs designed to improve the functional status of these patients. In our study, the mean 6MWD before and after the intervention was 567 and 581 m, respectively, thus giving a 14 m improvement post-intervention. These values are somewhat similar to the 6MWD in the general population as reported by Casanova *et al*. Compared with studies involving exercise programs for patients with chronic diseases, the 6MWD in our study is higher overall, with less improvement after physical rehabilitation. For example, Ballet *et al*. reviewed 15 studies that used the 6MWT to evaluate outpatient cardiac rehabilitation programs for patients with coronary artery disease (CAD) [16]. The pre-cardiac rehabilitation 6MWD ranged from 283 to 485 m, with a mean increase of 60 m post-rehabilitation. In COPD patients, a randomized clinical trial by Greulich *et al*. of 61 patients showed that a 3-month individualized training program increased the 6MWD by 32 m from 407 to 439 m [21]. A study of 26 patients on hemodialysis, who participated in combined aerobic and resistance training for 10 weeks, reported an increase of 6MWD from 440 to 480 m [22]. One explanation for the greater 6MWD found in our study is the relatively younger age of our patient population compared with patients with chronic diseases, with the majority of patients in this study being under the age of 50 years.

The only reported study that used 6MWD as an outcome measure of rehabilitation after solid organ transplant is by Munro *et al*., which demonstrated that a 3-month rehabilitation program for 36 lung transplant patients resulted in an increased of 6MWD from 451 m at 1 month post-transplant to 543 m at 3 months post-transplant [23]. The rehabilitation program described consisted of 1-hour group exercise training classes on an outpatient basis 3 days per week, combined with educational sessions via a multidisciplinary approach. Compared with our study, the increase in the 6MWD in that study was much larger. However, this could be partly attributed to the natural history of recovery from the transplant, given that post-transplantation rehabilitation is part of the standard of care, and there was no control group. Compared with our study, patients in the Munro *et al*. study were more supported throughout the program, as they were seen by the healthcare team at least three times per week. In addition, they also received relevant health education, which probably had a significant positive impact on their post-transplant self-care and overall health outcomes.

The improvement in 6MWD for the majority of participants in our study suggests that physical activity through AVG has the potential to improve cardiovascular and functional outcomes in RTRs. We did not find a correlation between the number of days used and the magnitude of quantitative improvement in the 6MWD. However, an increase specifically in the magnitude of 6MWD may be influenced not only by the amount of time spent on the AVG exercise system, but also by factors such as the participant’s baseline cardiovascular fitness and functional status and mobility, and the presence of other comorbidities. Larger studies may be helpful in elucidating the correlation between the amount of use of the AVG system and improvements in cardiovascular outcomes.

Although a number of studies have shown that there is generally an increase in the 6MWD that is attributable to learning effects, the 6MWTs in these studies were performed on the same day with an interval of 20 or 30 minutes [20,24]. By contrast, the 6MWTs in our study were completed by participants in a single attempt on different days at least 8 weeks apart. Although it is still possible that there may be a learning effect on the 6MWD, it is likely to be minimal in the setting of this study, given the amount of time that has passed between the attempts.

Participants over the age of 45 years were found to spend significantly more time on the AVG system compared with younger patients, which may be the result of a more flexible schedule, as most of them were retired. Similarly, most of the younger participants who were not able to comply with the recommended exercise regimen or complete the study had employment and multiple other unforeseen commitments, which restricted the amount of time they could spend on the AVG system. Future studies should focus on patients in an age group with more flexible schedules and offer longer exercise programs. In addition, more frequent follow-ups may be beneficial in serving both as a reminder as well as motivation for participants to use the AVG system to exercise. Another consideration for future studies would be to include patients from other transplant populations, as exercise training can improve the health outcomes of those patients as well. For example, cardiovascular fitness in liver transplant recipients is reported to affect severity of fatigue and quality of life [25]. Exercise training is also recommended for heart transplant recipients, as muscle wasting and exercise intolerance are common concerns in these patients [26]. Lastly, the AVG system used in this study has the capabilities to records the time and duration of use. However, most of the participants were not able to set up this data-keeping function because of technical difficulties. Instead, they were asked to record their AVG usage in a journal form to be submitted at the end of the study. Future studies would benefit from such record-keeping intrinsic to the AVG system if a more technically complete set-up is attainable.

## Conclusion

Physical activity through AVG has the potential to improve cardiovascular fitness in RTRs, especially those over the age of 45 years. Further research involving larger and longer studies is required to corroborate the full health benefits of AVG in this patient population.

## Abbreviations

AVG: Active video gaming; BP: Blood pressure; CAD: Coronary artery disease; CKD: Chronic kidney disease; CVD: Cardiovascular disease; GFR: Glomerular filtration rate; HDL: High-density lipoprotein; QOL: Quality of life.

## Competing interests

IA received Grant support from Novartis Pharmaceuticals Canada Inc solely for this study and has no conflict of interest to report in relation to this study. None of the other authors have any conflict of interest to report.

## Authors’ contributions

DWYW contributed to the design of the study, participated in ethics submission and selection of participants, conducted part of the research, performed the data analysis, and wrote the original manuscript. LLS conducted part of the research, facilitated data collection, participated in selection of participants, and read and revised the manuscript. SM conducted part of the research. ZM contributed to the design of the study, and participated in ethics submission and selection of participants. IA conceived and designed the study, participated in ethics submission, and provided approval for the final version. All authors read and approved the final manuscript.

## Authors’ information

Dorothy Wang, Department of Surgery, Multi-Organ Transplant Program, Dalhousie University, Room B02 Centre for Clinical Research, 5790 University Ave., Halifax, NS, Canada B3H 1 V7. Laura L. Sills, Department of Surgery, Multi-Organ Transplant Program, Dalhousie University, Room B02 Centre for Clinical Research, 5790 University Ave., Halifax, NS, Canada B3H 1 V7. Sara MacDonald, Department of Physiotherapy, Dalhousie University, Physiotherapy Department, 4th Floor Dickson Building, 5820 University Ave, Halifax, NS B3H 1 V8. Ziv Maianski, Department of Surgery, Multi-Organ Transplant Program, Dalhousie University, QEII Health Sciences Center, Office 6–291 Victoria Building, 1276 South Park Street, Halifax, NS, Canada B3H 2Y9. Ian Alwayn, Department of Surgery, Multi-Organ Transplant Program, Dalhousie University, QEII Health Sciences Center, Office 6–291 Victoria Building, 1276 South Park Street, Halifax, NS, Canada B3H 2Y9.
